# Mobile intron RNA from a bacterial predator accumulates in dead archaeal cells

**DOI:** 10.1038/s41598-026-51721-6

**Published:** 2026-05-07

**Authors:** Jana Kizina, Almud Lonsing, Jens Harder

**Affiliations:** Max Planck Institute for Marine Microbiology, Bremen, Germany

**Keywords:** Intron group I, Extracellular RNA, Mobile genetic element, Evolution, Microbiology, Molecular biology

## Abstract

Introns in prokaryotes are classified as mobile genetic elements normally located within essential genes. Their RNA transcripts are functional if spliced by the intron´s ribozyme activity. The excised intron RNA is assumed to proliferate by leaving the host cell and being integrated into foreign genomes. Here we show the presence of a bacterial intron´s RNA in archaeal cells. The predatory ultramicrobial epibiont *Candidatus* Velamenicoccus archaeovorus contains a group I intron in its 23 S ribosomal RNA gene. In search for predation evidence, we visualized the intron RNA by *in situ* hybridization and observed it in dead cells of the filamentous archaeon *Methanothrix soehngenii* in a slowly growing methanogenic enrichment culture on limonene. Transcriptome analysis detected a ratio of one unspliced transcript or intron molecule per 20,000 mature 23 S rRNA molecules. Our study visualized that spliced intron RNA can leave the host and enter foreign cells. Spliced intron RNA adds to extracellular RNA, so far known to function as intercellular signaling molecules and metabolic inhibitors.

**Supplementary Information:**

The online version contains supplementary material available at 10.1038/s41598-026-51721-6.

## Introduction

Introns are genetic elements that are self-splicing. Prokaryotic introns are often mobile^1^. Dispersal into novel prokaryotic hosts – a transposition - is currently considered to require vectors, phages for group I introns^1^ and plasmids for group II introns^2^. Structural defects encoded in introns result in loss of motility and degenerated introns^3^. Introns are often observed in predatory bacteria, usually located in essential genes. One of them is *Ca.* Velamenicoccus archaeovorus. In this study, we investigated the presence of the intron RNA in cells of an anaerobic enrichment culture using *in situ* hybridisation.

In microbial foodwebs, the primary consumers - chemoheterotrophic bacteria - are predated by phages, predatory bacteria and small protists. In anoxic systems, eukaryotes are very rare^4^. Necromass, the dead biomass after predation, is recycled by microorganisms. In closed systems, this anaerobic microbial loop can result in the accumulation of predatory bacteria. One example is a slowly growing methanogenic enrichment culture on the natural hydrocarbon limonene maintained by one transfer per year since over twenty years^5^. A 10% vol/vol inoculum produces methane over two years. The culture is dominated by an ultramicrobacterium with an epibiontic lifestyle, *Ca.* Velamenicoccus archaeovorus. Limonene degradation is performed by a *Syntrophobacteraceae* bacterium, and several methanogenic archaea including the acetoclastic filamentous *Methanothrix soehngenii* use the syntrophic products for methanogenesis^6^. The enrichment culture also contains several bacterial species specialized in fermenting necromass-derived compounds^6^. The predatory lifestyle of *Ca.* Velamenicoccus archaeovorus was inferred from individual cells in *Methanothrix soehngenii* filaments that were dead, indicated by the absence of 16 S ribosomal RNA, a DEAD appearance in LIVE/DEAD staining and less cell content in images. DNA and lipids were still detectable^5^. These cellular damages suggested a transfer of molecules from the predator into the prey cell. We selected an intron RNA as target molecule, based on its potential mobility and the sensitive technology to visualize nucleic acids in cells.

*Ca.* Velamenicoccus archaeovorus has a group I intron inserted into its 23 S ribosomal RNA gene at position 1,917 (referring to *Escherichia coli*)^5^. Group I introns are ribozymes that enable host life by splicing an intron RNA from the primary transcript, thereby producing mature transcripts, e.g. 23 S rRNA. In group I introns, a homing endonuclease encoded in the intron is expected to damage DNA, and the cellular repair mechanism is involved in the integration of the intron DNA into other genome sites^7^. Intron RNA molecules are rapidly degraded in eukaryotic cells, but can form stable circular RNA molecules with prolonged life time^8^. Group II introns disperse via their RNA molecules and a reverse transcriptase in a process called retrohoming. In the genome of *Ca.* Velamenicoccus archaeovorus, a retron-type RNA-directed DNA polymerase (reverse transcriptase) is encoded between a gene duplication far away from the intron. We imagined that intron RNA may enter the victim cell during the predation process as part of the intron proliferation. This hypothesis was tested in an *in situ* hybridization study, supported by reanalysis of a previously published RNA sequence data set.

## Results

**Fig. 1:**
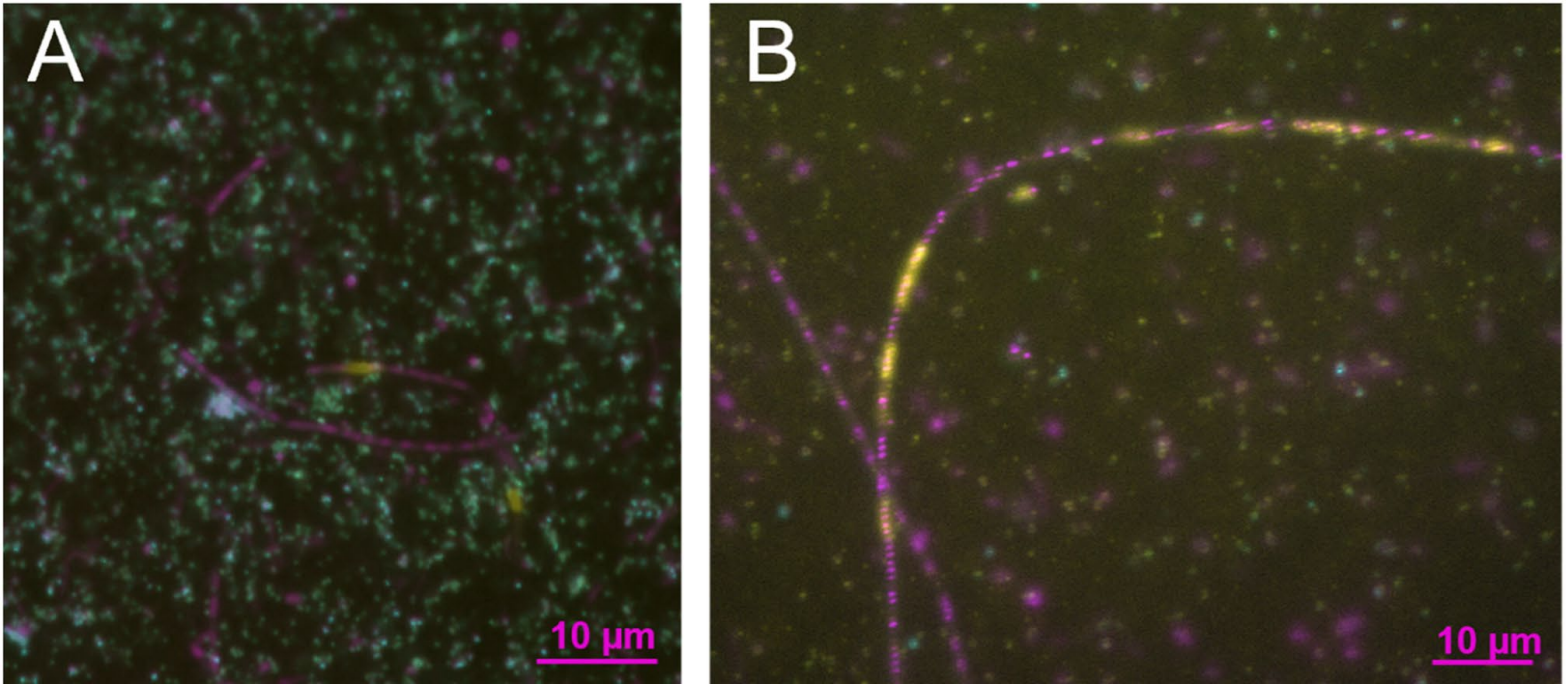
*In situ* detection of intron RNA in Methanothrix soehngenii. Light detection by epifluorescence microscopy (**A**) and super-resolution structured-illumination microscopy (**B**) revealed the presence of intron RNA detected by a mix of three intron probes (yellow), 16 S rRNA of the predator *Ca.* Velamenicoccus archaeovorus cells detected by probe OP-565 (turquoise) and DNA stained with DAPI (violet). *Ca.* Velamenicoccus archaeovorus cells are very small cocci and *Methanothrix soehngenii* cells are filaments of large cells. Some cells in the filament are dead, as indicated by the absence of DNA. A green color results from the mixing of turquoise and yellow, the presence of 16 S rRNA and intron RNA molecules in *Ca.* Velamenicoccus archaeovorus. Images originated from different cultures. The scale bar is 10 μm. For more details, see supplementary information.

**Fig. 2:**
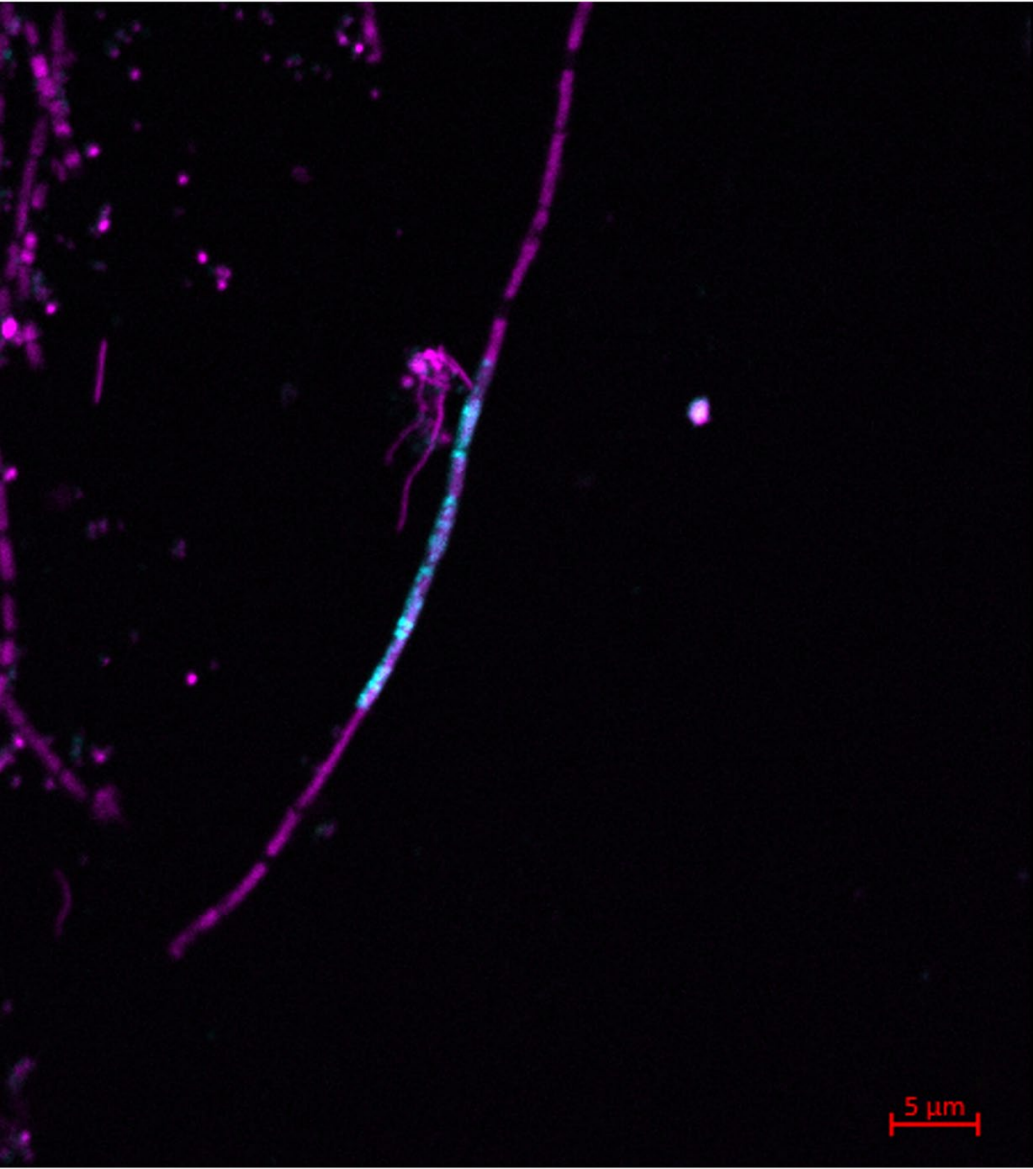
*In situ *detection of intron RNA in *Methanothrix soehngenii*. Image of one layer obtained by confocal laser scanning microcopy. Epiluorescence signals originate from DAPI (violet), CARD-FISH detection of intron probes hen1-2235 and hen3-2538 (torquoise) and the reverse complement probe hen2-rc2309 (yellow) that was expected to not show binding to the intron. *Methanothrix soehngenii* are filaments of large cells. The scale bar is 5 μm. For the overlay image, all epifluorescence channel settings were set to 0-150 greyscale intensity levels. The camera delivers 8 bite images with 255 grayscale intensity levels. For more details, see supplementary information.

We designed three horseradish-peroxidase labelled oligonucleotide probes (20 – 23-mer) for catalyzed reporter deposition-fluorescence *in situ* hybridization (CARDFISH) experiments and used these probes single or in mixes with helper oligonucleotides. In double hybridization experiments with probes for the intron and the 16 S rRNA of *Ca.* Velamenicoccus archaeovorus, the intron was detected in small cells identified as *Ca.* Velamenicoccus archaeovorus by a green color as result of the presence of intron RNA (yellow color) and 16 S rRNA (turquoise color) and in cells of large *Methanothrix soehngenii* filaments (Fig. 1, Suppl. Figure 1,2). The negative control of CARD-FISH experiments - a probe with a reverse complement sequence - did not yield specific hybridization signals. As positive control served intron probes that had other binding sites (Fig. 2, Suppl. Figure 3). The frequency of *Methanothrix soehngenii* cells with intron RNA was very low. Individual *Methanothrix soehngenii* cells are well isolated by a matrix and spacer plugs^9^. The position of spacer plugs appears in epifluorescence images as non-stained region separating cells. Single filaments in the enrichment contained viable and dead cells^5^. With the intron probes, we observed that intron RNA was only detected in dead cells that did not contain detectable amounts of archaeal 16 S rRNA (Supp. Figure 4, 5). In these cells, DNA was still detected (Fig. 1). Sometimes viable cells were neighboring, confirming an isolation by spacer plugs which can be seen as passive defense system avoiding the death of the whole filament (Supp. Figures 4, 5).

To find independent evidence of the existence of intron RNA molecules in the culture, we investigated the previously published metatranscriptome^5^. Epibiontic *Ca.* Velamenicoccus archaeovorus, large cells, filaments and aggregates were collected in a 10 000 Svedberg (S) pellet^5^. The transcriptome containing 57 878 109 reads and was not depleted of rRNA molecules. The sequence data revealed a mean coverage of the intron of 14. In comparison, the mature 23 S rRNA had a mean coverage of 263 770. The intron-exon borders were also detected in individual reads of the transcriptome, with 23 and 20 reads that bridged the borders on the start and end of the intron, respectively, and included 5 or more bases on each side of the border (Suppl. Figure 6–9). Thus, only a tiny fraction of the transcripts was not spliced by the ribozyme.

## Discussion

Intron mobility within a strain has been characterized in great detail^1–3^. Mobility of introns between species has been inferred from gene sequences that demonstrated incongruence among phylogenetic trees and introns in non-homologous lineages. Our observations provide an *in vivo* evidence of the mobility of intron RNA between cells of different organisms. This is a prerequisite for a transposition into a foreign species, for a horizontal gene transfer.

The detection of intron RNA enlarges the variety of molecules in extracellular RNA. Extracellular RNA of microorganisms comprises many small RNA molecules that act by inhibiting transcription and, in consequence, metabolic activity^10^. In addition, extracellular RNA informs the environment on the presence of the producing microbe, e.g. the transfer-messenger RNA encoded by *ssrA* of *Aliivibrio fischeri* reduces the activity of the host defense system of the Hawaiian bobtail squid *Euprymna scolopes*, thereby enabling a full symbiosis with bioluminescence^11^. This interaction was also experimentally verified by *in situ* detection of the extracellular RNA molecule. The evolution of extracellular RNA molecules for many biological functions may be entailed with the late evolution of deoxyribonucleotide synthesis^12–14^.

The detection of intron RNA in *Methanothrix soehngenii* cells that lack 16 S rRNA can be explained by circularization of intron RNA. Circular RNA is a post-splicing product of intron excision of group I introns^15^ and resistant to RNA degradation by exoribonucleases^16^. Transposition into a foreign genome requires reverse transcriptase activity^17^. The retron-type RNA-directed DNA polymerase encoded in *Ca.* Velamenicoccus archaeovorus was detected as protein in the metaproteome of the limonene enrichment culture^5^. Transmission electron micrographs^5^ showed the absence of membranes and an open contact of cytoplasm between predator and victim cells. If the reverse transcriptase also moved into the victim cell, transposition may have a chance to proceed. Our observations together with previous observations, e.g. the reversibility of splicing, suggest that group I introns can transpose via an RNA-based transposition pathway.

## Methods

### Cultivation

Anaerobic cultivation techniques were applied^18^. One liter of freshwater medium contained 1.0 g of NaCl, 0.4 g of MgCl_2_ × 6 H_2_O, 0.1 g of CaCl_2_, 0.5 g of KCl, 0.2 g of KH_2_PO_4_, 0.25 g of NH_4_Cl, and 0.2 g of NaSO_4_ in pure water. After autoclaving, 1 ml of the following sterile solutions was added to 1 l of medium: chelated trace element mixture (per liter of distilled water: 2100 mg of FeSO_4_ × 7 H_2_O, 60 mg of H_3_BO_3_, 1.0 g of MnCl_2_ × 4 H_2_O, 0.38 g of CoCl_2_ × 6 H_2_O, 0.24 g of NiCl_2_ × 6 H_2_O, 2 mg of CuCl_2_ × 2 H_2_O, 0.29 g of ZnSO_4_ × 7 H_2_O, 72 mg of NaMoO_4_ × 7 H_2_O, and 7.8 g of Na_2_EDTA, pH 6.0), selenite-tungstate solution (per liter: 0.4 g of NaOH, 32 mg of Na_2_WO_4_ × 2 H_2_O, 24 mg of Na_2_MoO_4_ × 2 H_2_O, and 6 mg of Na_2_SeO_3_ × 5 H_2_O), vitamin solution (4 mg of 4-aminobenzoic acid, 2 mg of D-(+)-biotin, 10 mg of nicotinic acid, 5 mg of calcium D-(+)-pantothenate, 15 mg of pyridoxin hydrochloride, 4 mg of folic acid, and 1.5 mg of lipoic acid in 100 ml of 10 mM NaH_2_PO_4_, pH 7.1), cyanocobalamin solution (5 mg L^− 1^), thiamine solution (10 mg of thiamine hydrochloride in 100 ml of 25 mM NaH_2_PO_4_, pH 3.4), and riboflavin solution (2.5 mg in 100 ml of 25 mM NaH_2_PO_4_, pH 3.2). After addition of sterile stock solutions − 2 mL of 0.5 M cysteine, 2 mL of 1 M sodium acetate and 30 mL of 1 M NaHCO_3_ - the pH was adjusted to 7.0 with sterile 2.0 M HCl.

The methanogenic enrichment culture was maintained with an annual transfer of 10% (vol/vol) inoculum. The cultures contained 300 mL freshwater medium, 0.3 mL of 200 mM FeS suspension, 30 mL 2,2,4,6,8,8-heptamethylnonane (HMN), and 1.5 mL of *R*-(+)-limonene under an oxygen-free N_2_–CO_2_ atmosphere (90:10, vol/vol, < 7 ppm O_2_) in 500-mL borosilicate bottles. Incubation occurred on an orbiting shaker with 60 rpm at 28 °C^5^.

### Cell separation and RNA extraction

Biomass was obtained by differential centrifugation. Large and aggregated cells were pelleted in a SW28 ultracentrifuge rotor (Beckman, Palo Alto, CA) at 7600 rpm (7643 *g*) for 20 min (10,000 Svedberg [S]). The 10 kS cell pellet was resuspended in 1 mL of 10 mM Tris, 1 mM EDTA, pH 8.0 (TE). RNA was extracted using the RNA PowerSoil Total RNA Isolation Kit (Qiagen, Hilden, Germany). RNA sequencing was performed by the Max Planck-Genome-centre Cologne, Germany (https://mpgc.mpipz.mpg.de/home/) using NEB Next Ultra RNA kit (New England Biolabs, Ipswich, MA) for the production of an Illumina-compatible library and Illumina HiSeq (Illumina, San Diego, CA) (150 bp reads; 57,878,109 reads revealing 8,918,223,863 bases were deposited at NCBI SRR30230645)^6^.

### *In situ* hybridization

Catalyzed reporter deposition-fluorescence *in situ* hybridization (CARD-FISH)^19^ was used targeting 16 S ribosomal RNA and intron RNA. Living *Methanosaeta* cells were identified with probe ARCH915 (GTGCTCCCCCGCCAATTCCT)^20^. *Ca.* Velamenicoccus archaeovorus probe OP3-565 (TACCTGCCCTTTACACCC)^21^ was used together with manually designed partially degenerated helper oligonucleotides H548-A (AATAAATCCGAGTAACGC), H548-C (AATCAATCCGAGTAACGC), H583-TC (CTCCCCACTTGTCAGGCCGCC) and H583-CT (CCTCCCACTTGTCAGGCCGCC)^5^. The group I intron in the 23 S rRNA was targeted within the gene for homing endonuclease. Probes and flanking helper oligonucleotides were designed using Primer3 v. 4.0.0 (http://sourceforge.net/projects/primer3/). Specificity was tested against the metagenome datasets of the enrichment culture^6^. Probes were hen1-2235 (CCGCCAAGTAGTAGCCGATT), hen2-2309 (AACTTTCCACGGTGACTTGT), hen3-2538 (TCTTTATCATTTCTGCCAGTTCG) and the reverse complement hen2-rc2309 (ACAAGTCACCGTGGAAAGTT). Helper were Hhen1-2210 (TCTGGTTTCATAAAGACCTCCTTCT), Hhen1-2255 (TCCTTCTCCGTCGGCAAAAC), Hhen2-2288 (AGTCTTGCCGTTGTCTGAAGG), Hhen2-2329 (CTGGGAGATGTTGAAACAAAGAGA), Hhen3-2516 (CAGAATTTCGCAAAATCCCGTT), Hhen3-2561 (GCTACTCTTAAGATGTTCCCCCT). For intron detection, a formamide concentration of 20% was used at concentrations of 0.17 ng/µl for each probe and helper. One mL of culture was fixed with formaldehyde (1.3% [wt/vol]) for 60 min at 21 °C. Five µL of fixed culture were dried on a glass slide and then rinsed with 1 ml of phosphate buffered saline (PBS, pH 7.4). Cells were embedded in 0.1% [wt/vol] low-melting agarose and air dried. Permeabilization was performed for 60 min at 37 °C by lysozyme (10 mg/mL) and proteinase K (200 µg/ml) for 2 min at 21 °C. Washing steps were performed as described^19^. Endogenous peroxidases were inactivated with 0.1 M HCl for 1 min at 21 °C, a washing with 1x PBS, and 3% H_2_O_2_ for 10 min at 21 °C. The probes were hybridized for 160 min at 46 °C, followed by incubation with Alexa448 or Alexa594-tyramide conjugates at 46 °C for 45 min in the dark. After DNA staining with 4′,6-diamidino-2-phenylindole (DAPI, 1 µg/mL), cell preparations were embedded in Citifluor-Vectashield antifading medium (4:1 [vol/vol]). Images were obtained with an epifluorescence microscope (Zeiss Axiophot with camera MRm and HBO100 light source using Zeiss Axiovision). Confocal laser scanning microscopy (CLSM) and super resolution structured illumination microscopy (SR-SIM) were performed using a Zeiss LSM 780 equipped with a ELYRA PS.1 module. Zeiss Zen 3.11 was used to analyse the images (Zeiss, Oberkochen, Germany).

### Sequence analysis

RNA reads (NCBI: SRR30230645) were trimmed with BBDuk to > Q30 and longer than 30 nt. Coverage of reads mapping with at least 99% identity was determined using BBMap and *Ca.* Velamenicoccus archaeovorus (NCBI CP019384) or partial sequences thereof (300 nt for exon/intron border, intron sequence). Both calculations were performed within Geneious (Dotmatics, Boston, MA) with the default parameters within Geneious, except for the ones stated. Reads covering the borders between intron and exon were quantified by manual inspection of mapped and aligned reads (150 bp) to aforementioned partial sequences.

## Supplementary Information

Below is the link to the electronic supplementary material.

Supplementary Material 1

## Data Availability

The dataset reanalysed during the current study is available from the NCBI repository [SRR30230645].
