# Supplementary material for: Mobile intron RNA from a bacterial predator accumulates in dead archaeal cells
Source: Sci Rep. 2026 May 7;16:14654. doi: 10.1038/s41598-026-51721-6 (PMC13153191; doi:10.1038/s41598-026-51721-6)
Supplement: Supplementary file 1 — Supplementary Material 1 [file 41598_2026_51721_MOESM1_ESM.pdf]

Supplementary information

Mobile intron RNA from a bacterial predator accumulates in dead archaeal cells

Jana Kizina[*], Almud Lonsing[*], Jens Harder[#]

Max Planck Institute for Marine Microbiology, Bremen, Germany

Scientific reports

Supplementary table 1. Parameters for microscopic images Figs. 1A, B, and 2.

Image	Fig. 1A	Fig. 1B	Fig. 2
	epifluorescence microscope	SR-SIM	CLSM
microscope	Zeiss Axiophot	Zeiss LSM 780 with Elyra PS.1	Zeiss LSM 780
objective	Plan-Neofluar 100x/1.3 Oil	Plan-Apochromat 63x/1.4 Oil	Plan-Apochromat 63x/1.4 Oil
camera/detector type	AxioCamMRc	Andor iXon DU 885	PMT
light source	Zeiss HBO100	laser	laser
image aquisition	sequential	sequential	sequential
channel 1	Zeiss filter set 02		
excitation wavelength	365 nm	405 nm	405 nm
beam Splitter	395 nm		
detection range	> 420 nm	BP 420-480 nm	410-508 nm
exposure time	30 ms		
channel 2	Zeiss filter set 10		
excitation wavelength	450-490 nm	488 nm	488 nm
beam Splitter	510 nm		
detection range	515-565 nm	502-538 nm	490-588 nm
exposure time	390 ms		
channel 3	Zeiss filter set 15		
excitation wavelength	540-552 nm	561 nm	561 nm
beam Splitter	580 nm		
detection range	> 590 nm	573-613 nm	585-734 nm
exposure time	500ms		
other information		3 pattern angles for SIM	

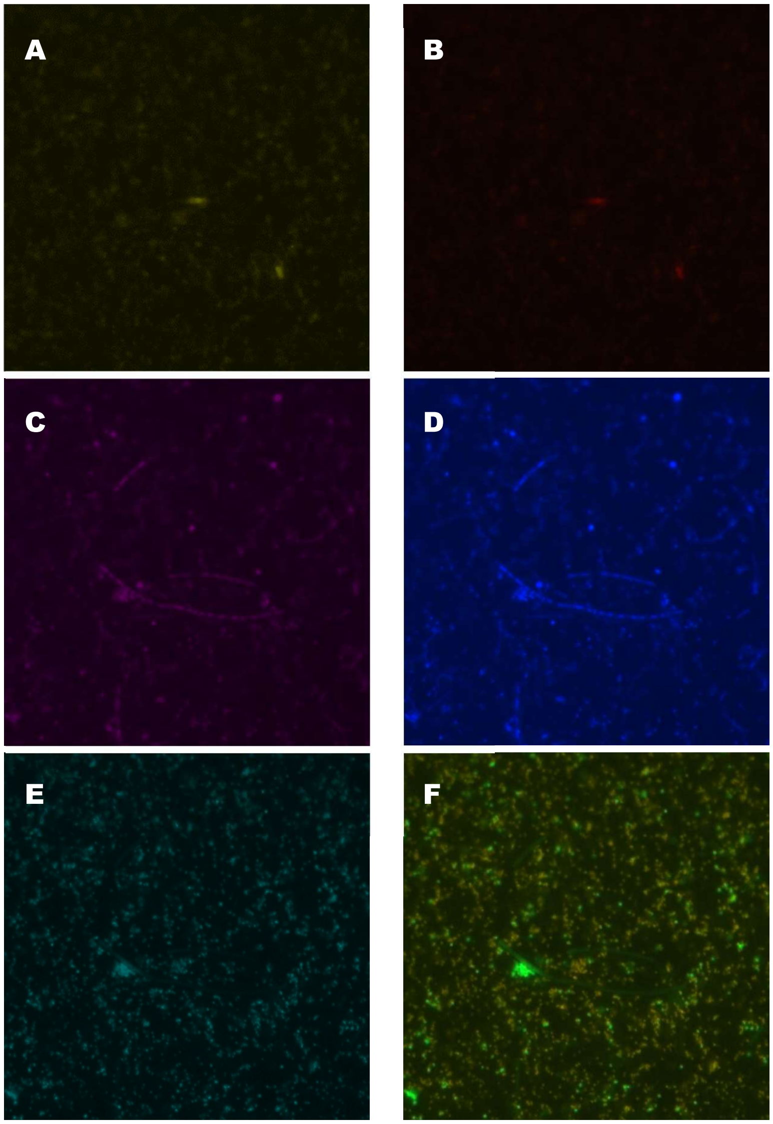

Supplementary figure 1. *In situ* detection of intron RNA in *Methanothrix soehngenii* in a CARD-FISH dual probe experiment: single channel images (for an overlay image, see fig. 1A). Epifluorescence signals indicate the presence of intron RNA (A,B), DNA (C,D) and 16S rRNA of the predator *Ca.* Velamenicoccus archaeovorus cells (E,F). B, D and F are original color-coded images, A, C, and E have colors accessible for color-blind persons.

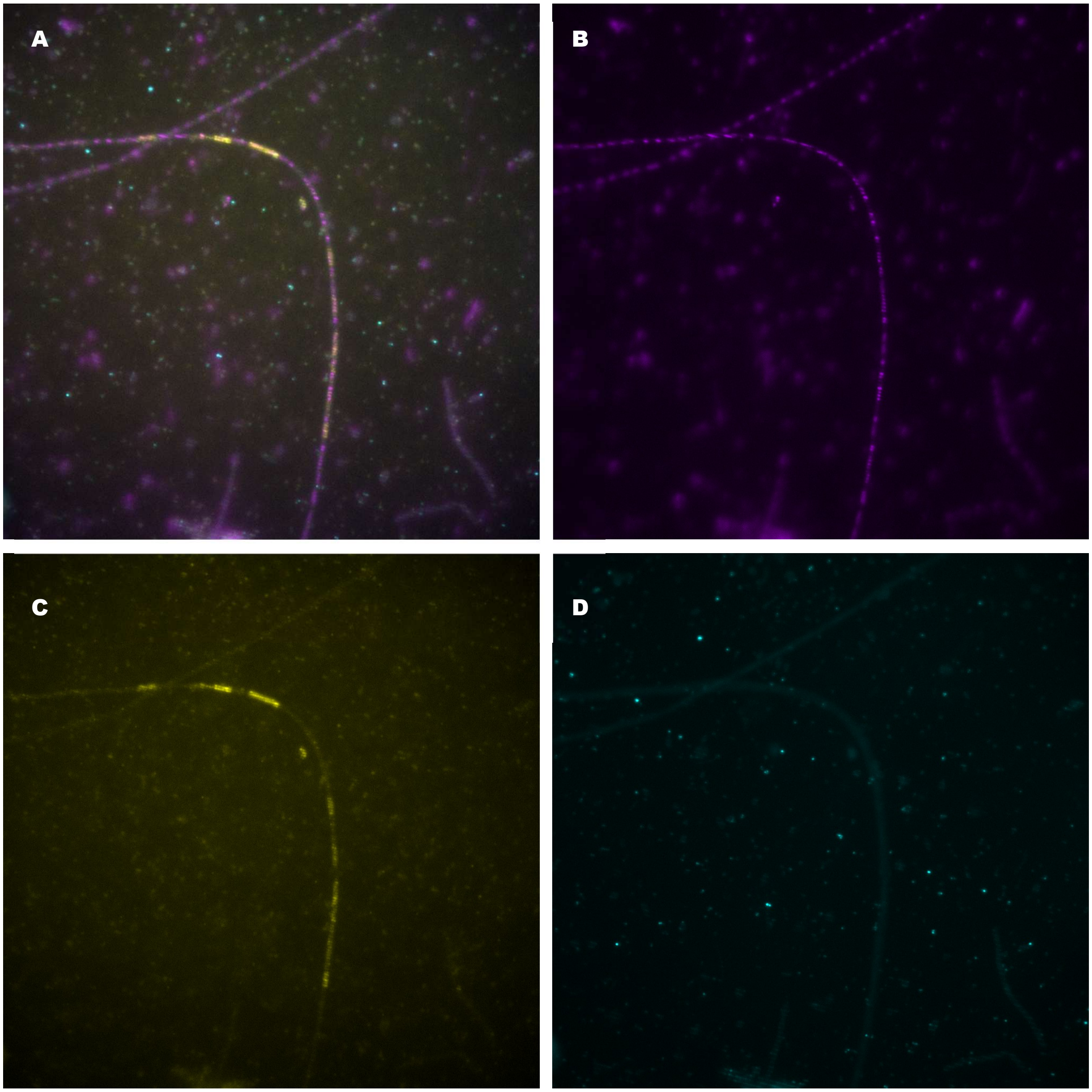

Supplementary figure 2. *In situ* detection of intron RNA in *Methanothrix soehngenii* in a CARD-FISH dual probe experiment: overlay and single channel images for one z-layer in a SR-SIM image (see also fig. 1B). Epifluorescence signals indicate the presence of DNA (violet, A, B), intron RNA (yellow, A, C), and 16S rRNA of the predator *Ca.* Velamenicoccus archaeovorus cells (torquoise, A, D).

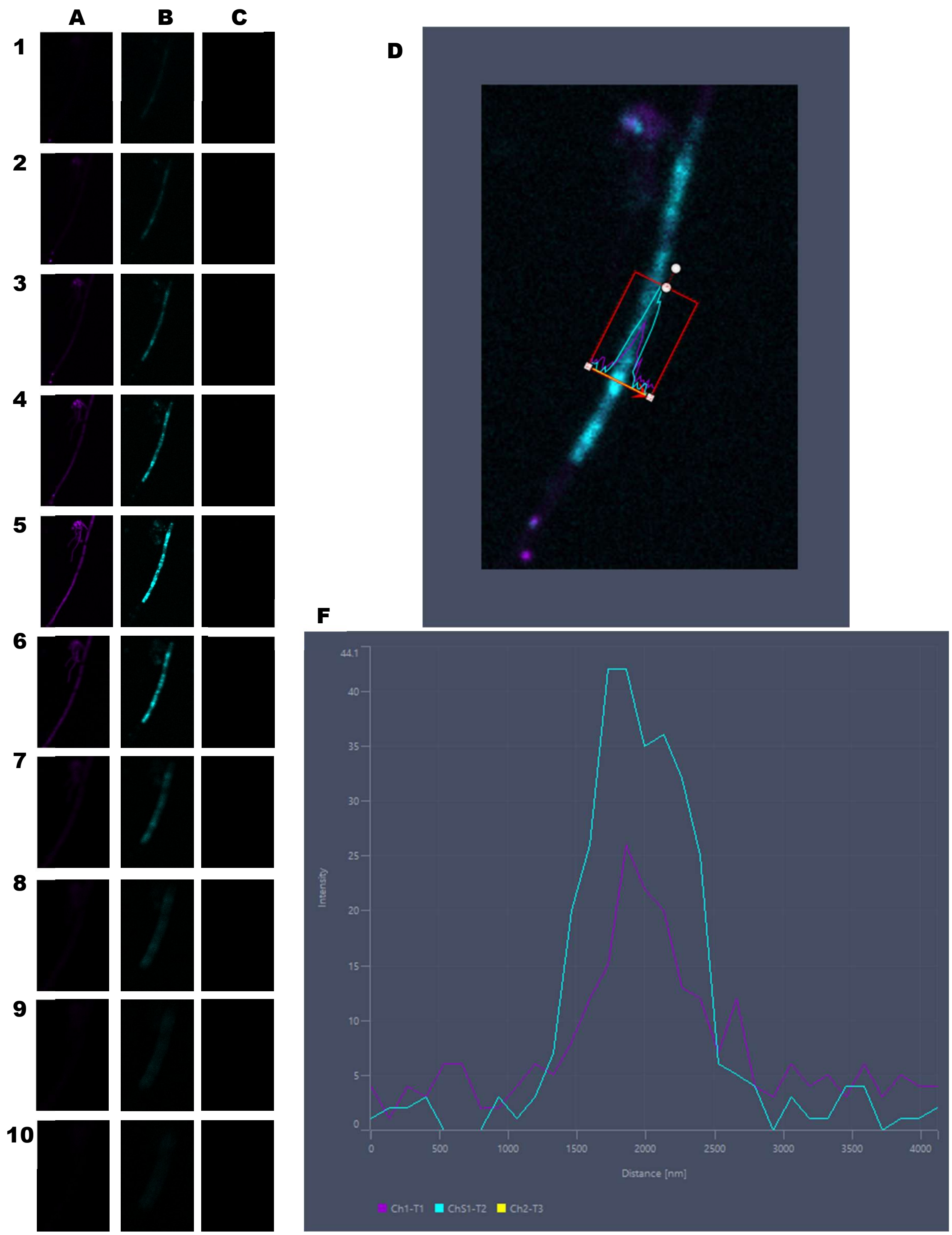

Supplement figure 3: *In situ* detection of intron RNA in *Methanothrix soehngenii*: individual images of ten z-layers detected in SR-SIM mode. Epiluorescent detection of DNA (violet, A), intron probes hen1-2235 and hen3-

2538 (turquoise, B) and the reverse complement probe hen2-rc2309 (yellow, C). The latter was expected to not show binding to the intron. E and F document the pixel intensity across a *Methanothrix soehngenii* filament cell.

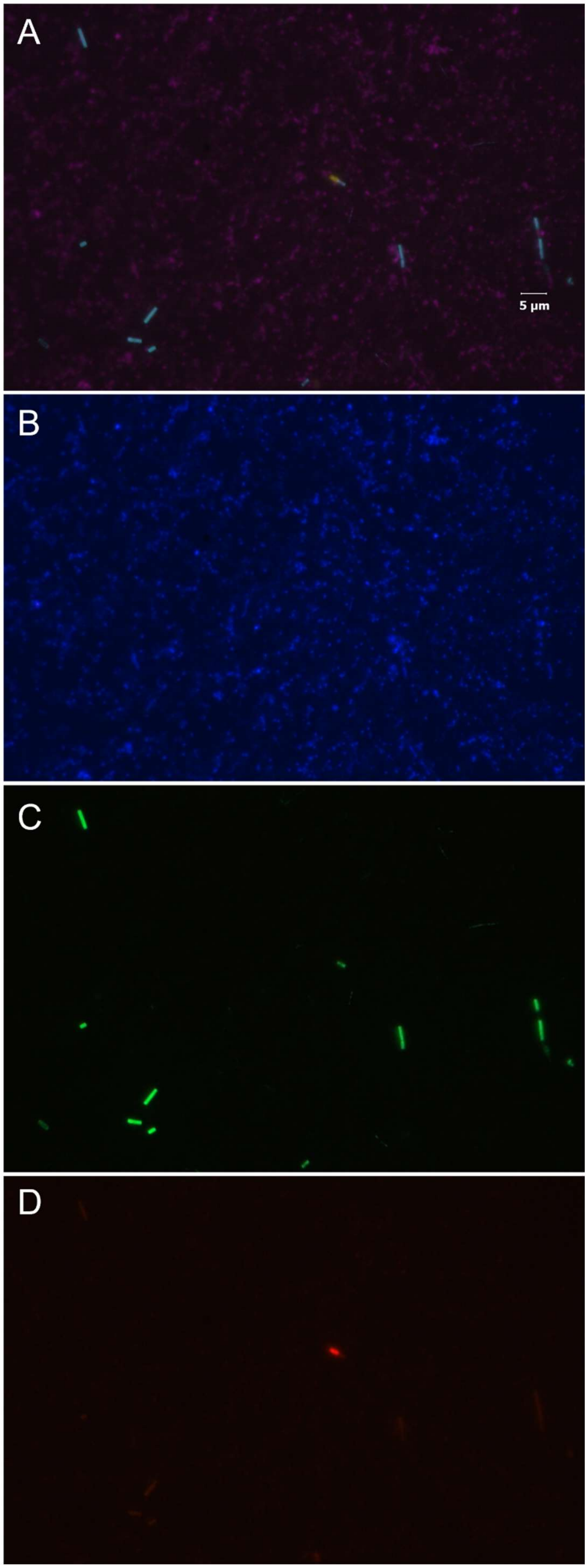
A
5 μm
B
C
D

Supplement figure 4: *In situ* detection of intron RNA in *Methanothrix soehngenii* in a CARD-FISH dual probe experiment: overlay and single channel images. Epiluorescence signals indicate the presence of DNA (DAPI (violet, A, blue, B)), archaeal 16S rRNA (probe ARCH-915 (tortoise, A; green, C)) and intron RNA (mix of three probes with helpers (yellow, A; red, D)). The epifluorescence images are shown in best-fit mode.

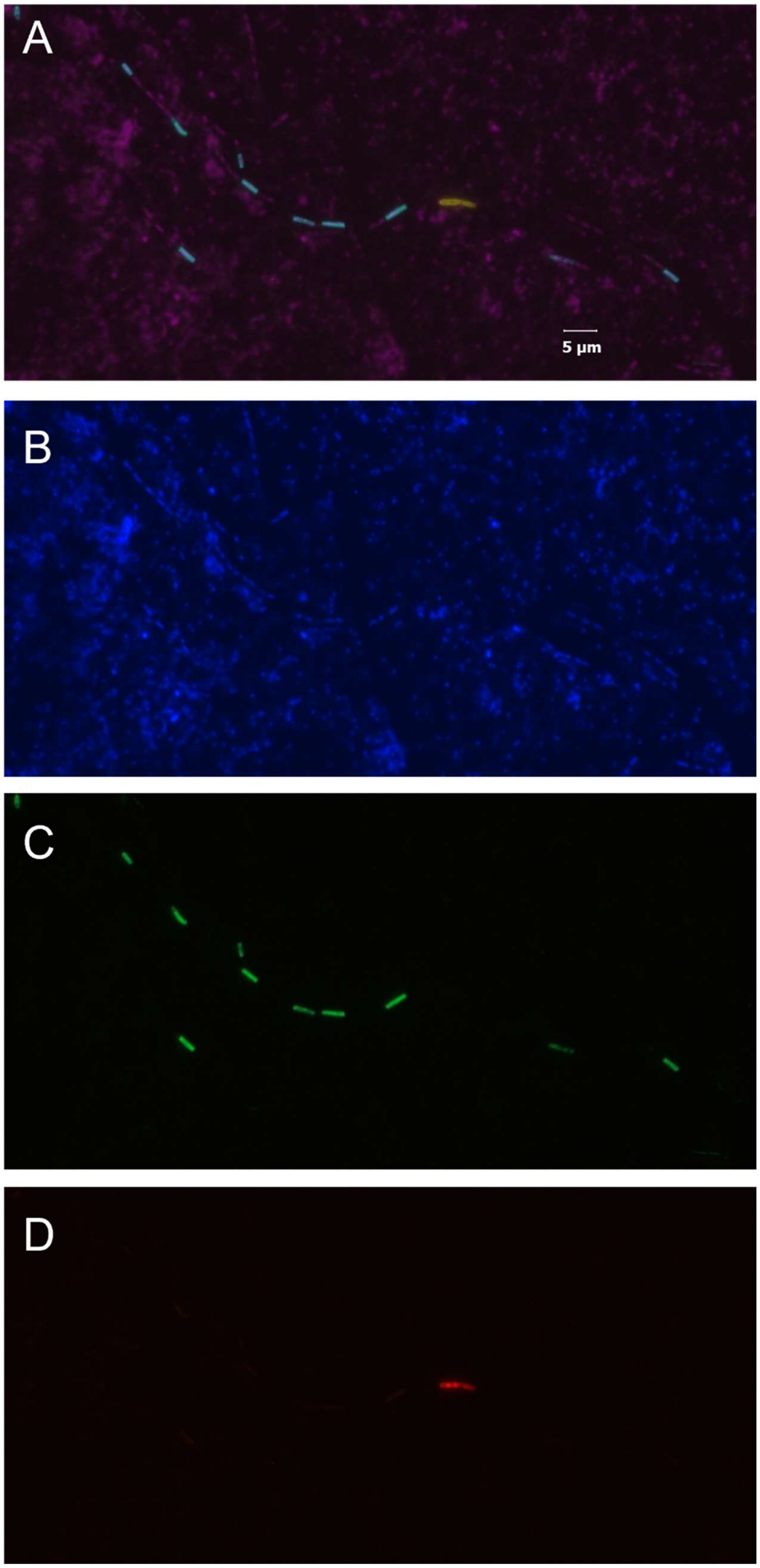

Supplement figure 5: *In situ* detection of intron RNA in *Methanothrix soehngenii* in a CARD-FISH dual probe experiment: overlay and single channel

images. Epiluorescence signals indicate the presence of DNA (DAPI (violet, A, blue, B)), archaeal 16S rRNA (probe ARCH-915 (tortoise, A; green, C)) and intron RNA (mix of three probes with helpers (yellow, A; red, D)). The epifluorescence images are shown in min-max mode.

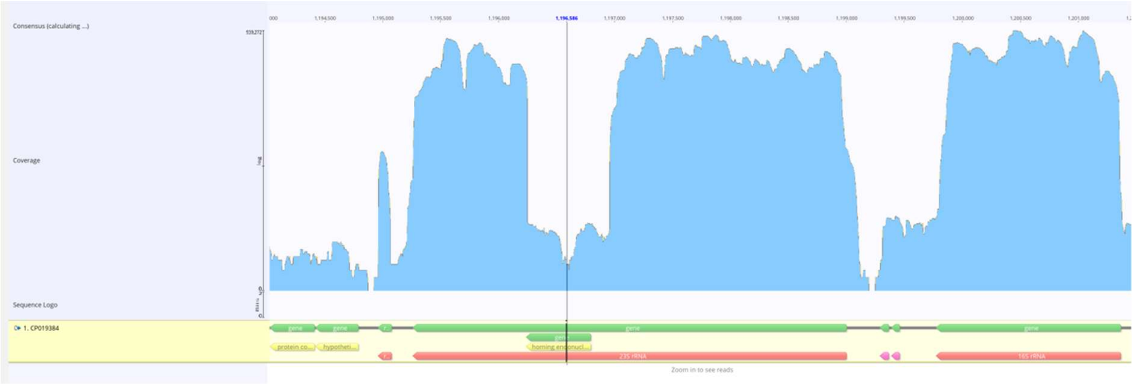

Supplementary figure 6. Visualization of read coverage of rRNA operon of *Ca.* Velamenicoccus archaeovorus (screen shot from Geneious). The line is in the middle of the intron sequence, within the open reading frame of the homing endonuclease. Note the logarithmic scale on the y-axis.

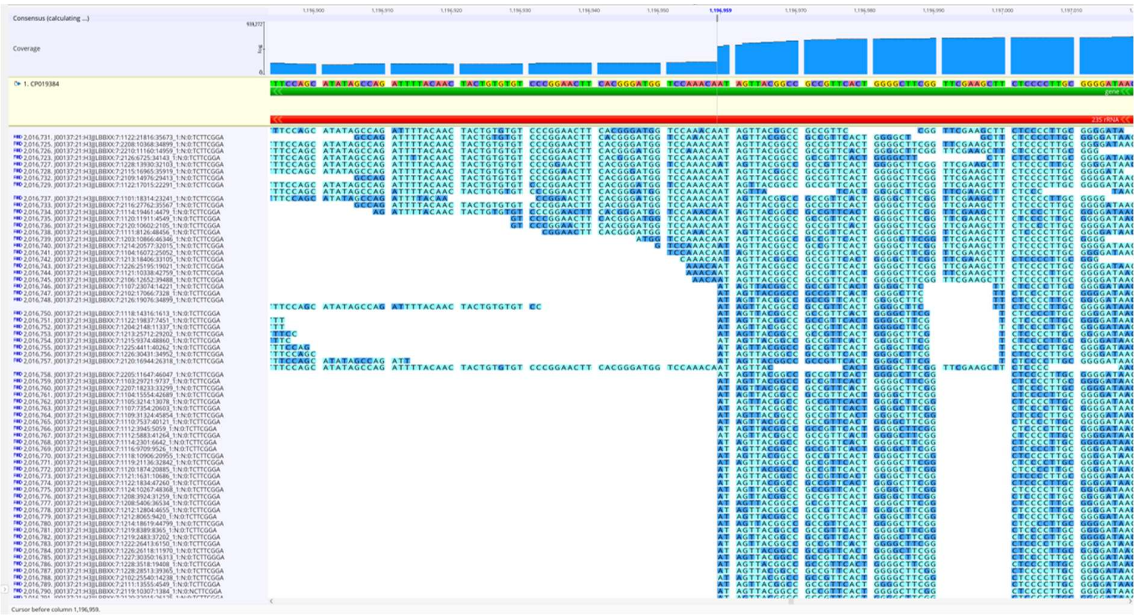

Supplementary figure 7. Partial read coverage of rRNA operon of *Ca.* Velamenicoccus archaeovorus. The sequence covers the start of the intron in the 23S rRNA gene (screen shot from Geneious).

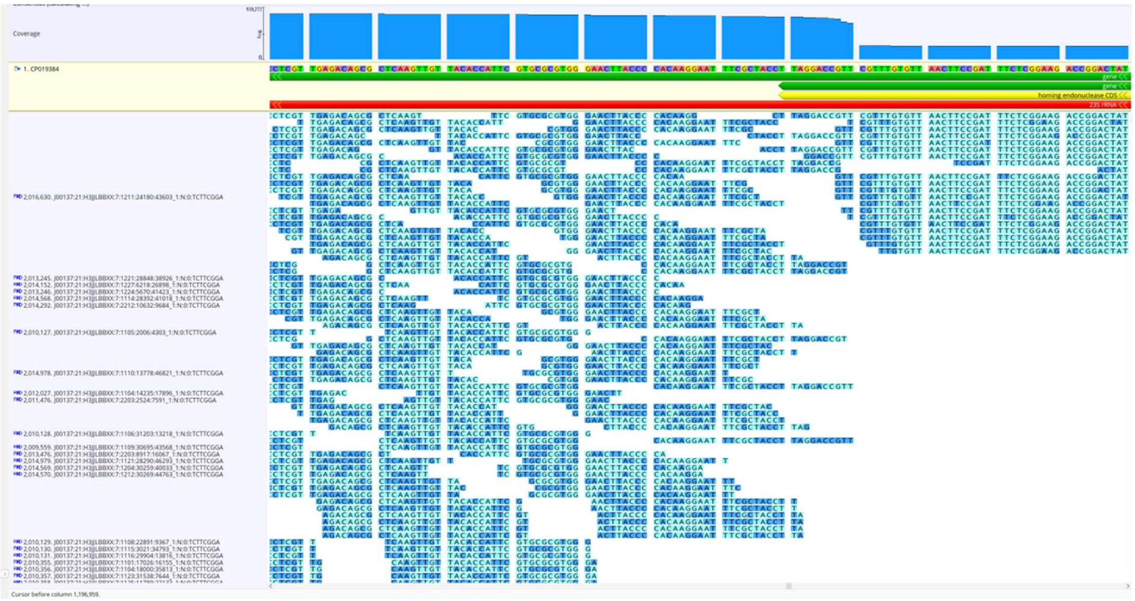

Supplementary figure 8. Partial read coverage of rRNA operon of *Ca.* Velamenicoccus archaeovorus. The sequence covers the end of the intron in the 23S rRNA gene (screen shot from Geneious).

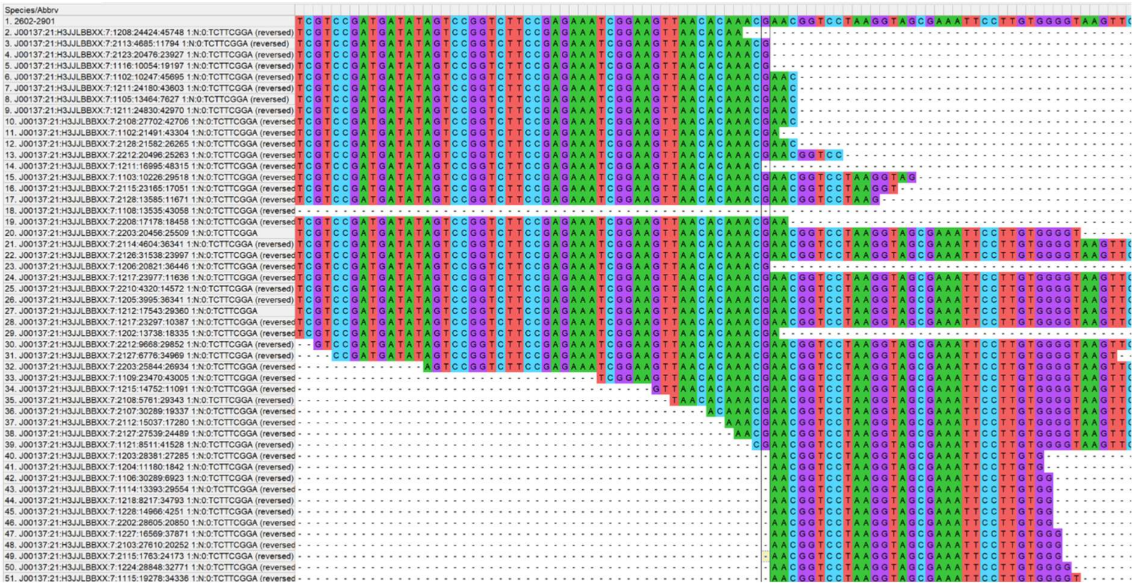

Supplementary figure 9. Alignment of mapped reads to partial rRNA operon of *Ca.* Velamenicoccus archaeovorus. Shown is the end of the intron with a frame around the last intron nucleotide (screenshot from MEGA12).
